# Does the diurnal cycle of cortisol explain the relationship between physical performance and cognitive function in older adults?

**DOI:** 10.1186/s11556-017-0175-5

**Published:** 2017-05-15

**Authors:** B. Dijckmans, J. Tortosa-Martínez, N. Caus, G. González-Caballero, B. Martínez-Pelegrin, C. Manchado-Lopez, J. M. Cortell-Tormo, I. Chulvi-Medrano, A. Clow

**Affiliations:** 1 Maastricht University, Maastricht, The Netherlands; 2 Universidad de Alicante (Facultad de Educación), Carretera San Vicente del Raspeig s/n., 03690 Alicante, Spain; 3 Neurology Unit, Hospital de San Vicente del Raspeig, Raspeig, Spain; 4 University of Westminster, San Vicente del Raspeig, UK

**Keywords:** Aging, Mild cognitive impairment, Dementia, Cognitive function, Physical activity, Cortisol, Chronic stress, HPA axis

## Abstract

**Background:**

Regular physical activity is a promising strategy to treat and prevent cognitive decline. The mechanisms that mediate these benefits are not fully clear but physical activity is thought to attenuate the harmful effects of chronic psychological stress and hypercortisolism on cognition. However, the circadian pattern of cortisol secretion is complex and it is not known which aspects are most closely associated with increased cognitive function and better physical performance. This is the first study to simultaneously measure cognitive function, the diurnal cycle of salivary cortisol and physical performance in older adults, without cognitive impairment (*n* = 30) and with amnestic Mild Cognitive Impairment (aMCI) (*n* = 30).

**Results:**

Regression analysis showed that better cognitive function was associated with better physical performance. A greater variance in cortisol levels across the day from morning to evening was associated with better cognitive function and physical performance.

**Conclusions:**

The results support the idea that a more dynamic cortisol secretion pattern is associated with better cognitive function and physical performance even in the presence of cognitive impairment, but our results could not confirm a mediating role in this relationship.

## Background

Mild cognitive impairment (MCI) refers to a state of cognitive deterioration that exceeds what is expected for normal aging but yet does not meet the criteria for diagnosis of dementia. The prevalence rate of MCI in people aged 70 years or older is around 14–18% [1]. Especially the condition of amnestic MCI (aMCI), in which memory loss is the predominant symptom, is likely to progress to AD and seems to be adequate for early treatment in order to decrease the social and economic impact of AD [2, 3]

Besides age, several clinical conditions [4] and genetic agents [5], psychological stress and consequent dysregulation of the hypothalamic pituitary (HPA) axis [6] is considered to be an important risk factor of cognitive impairment and dementia. Psychological stressors stimulate activity of the HPA axis which, over a prolonged period of time, can lead to changes in the circadian pattern of cortisol secretion, including increased circulating levels. Excessive levels of cortisol have been argued to be related with chronic psychological stress, decline in cognitive function and development of cognitive illness [6]. However, the circadian pattern of cortisol secretion is complex and it is still under debate which aspects are most closely associated with cognitive function. In general, the diurnal cycle of cortisol secretion is characterized by an increase in levels following awakening, showing a peak after around 30 min (the cortisol awakening response: CAR) and a subsequent decline over the remainder of the day [7]. Abnormal secretion patterns such as a flattening of both the CAR and the cortisol diurnal decline have been shown to have a negative impact on cognition in healthy older adults [8–10]. Furthermore, a greater CAR and a subsequent steeper fall have been also associated with greater brain plasticity [11].

In parallel, exercise has been shown to improve memory and executive function in healthy older adults [12] and regular physical activity has been shown to be protective against and beneficial for Mild Cognitive Impairment, dementia, and Alzheimer’s disease [12–15]. The mechanisms underlying these benefits remain unknown although it has been suggested that exercise-induced changes in the circadian pattern of cortisol secretion may be implicated [16, 17].

Although evidence is scarce, it has been suggested that higher participation in PA and higher physical performance may improve resilience to stress [18, 19], support recovery from acute stress [18], reduce the autonomic and adrenocortical stress response [20, 21], and protect against the neuroendocrine effects of psychological stress [18]. However, some inconsistencies in the literature examining the impact of exercise on cortisol secretion should also be noted [22]. These inconsistencies may partially be explained by the fact that studies looking at the effects of exercise programs on cortisol secretion had often measured cortisol at one time-point, not considering the complete diurnal cycle of cortisol [13]. More recently, some studies conducted with healthy older adults have shown a relationship between a more dynamic cortisol secretion pattern and physical performance [23–25]. Two other studies have considered the effect of physical activity on cognitive performance in people with aMCI [26, 27]. Baker et al. [26] analyzed cortisol using a one time-point measurement (ranging from 8:00 to 10:00), without controlling for time of awakening, which again might not be the most accurate measure. The study conducted by Tortosa-Martínez et al. [27] found that a 3-month exercise program increased the dynamics of cortisol secretion and improved executive function in people with aMCI.

Considering the evidence on the interactions between physical activity, levels of cortisol and cognitive function, it is plausible that dysregulation of HPA axis function is involved in the interaction between physical activity and cognitive function [16]. However, studies investigating the interactions between all three factors are sparse and do not allow yet for robust conclusions about the effect of PA on levels of cortisol and cognitive function [19, 28]. Thus, the aim of this study was to explore associations between cognitive function, the diurnal cycle of cortisol and physical performance in healthy elderly and elderly with aMCI. We hypothesized that dysregulation in aspects of the diurnal cycle of cortisol secretion would account for the relationship between physical performance and cognitive function in this population.

## Methods

### Sample and recruitment

We recruited 30 older adults diagnosed with aMCI according to Petersen criteria [29] from the Neurology Unit of the Hospital de San Vicente del Raspeig in Spain. Additionally, 30 healthy older adults were recruited by advertisement. They were excluded if they were diagnosed with any type of cognitive impairment or memory complaints. An age of at least 65 years was obtained for all participants in the study. Taking anticholinergic medication, suffering from physical, cardiovascular or sensorial limitations for doing exercise safely or displaying indications for severe apathy, delirium or agitation were additional exclusion criteria for both groups.

### Measures

#### Cognitive function

The Mini-Mental State Examination (MMSE) was used to measure general cognitive function and covers orientation, memory and attention of participants. The test is proven to be valid and reliable [30].

We performed the Trail Making Test (TMT) to assess visuoperceptual abilities, working memory, mental flexibility and executive functions [31]. The test consists out of a simple A-part (TMT-A) and a more complicated B-part (TMT-B).

#### Salivary levels of cortisol

Each participant was provided with five Salivette tubes (Sarstedt, Newton, NC, USA). Salivary samples had to be taken at awakening time, 30 min post awakening and at 12 p.m., 17 p.m. and 21 p.m. (COR1 to COR5, respectively). All participants received detailed one-to-one instruction about the study protocol with the lead researcher. During this session they could practice the saliva collection technique and ask questions about the protocol. Patients with aMCI were accompanied by a carer who could oversee the successful completion of all instructions. Participants were instructed to place the saliva sampling kits next to their bed before going to sleep. On the morning of saliva sampling participants were instructed to put the salivette dental swab from the correctly labelled salivette into their mouths at the specified times and gently chew on it until fully saturated. Subsequently, the swab had to be stored in the refrigerator inside the tube. Salivettes were then taken into the laboratory of the laboratory and centrifuged at 1000g per 2 min, and stored at -20° until further analysis. Concentrations of cortisol were measured in duplicates using a modification of the solid-phase radioimmunoassay technique (RIA; Coat-A-Count, Siemens Medical Solutions Diagnostics, Los Angeles, CA, USA). Radiation in the samples was determined using a Packard Cobra Auto Gamma Counter (Auto-Gamma 5000 series, Cobra 5005, Packard Instruments Company, Meriden, CT, USA). Intra and interassay coefficients of variance were below 10%.

To avoid bias participants were requested to avoid eating, drinking (except for water), smoking, or brushing their teeth 30 min prior to sampling. To assess adherence to the sampling regime, all participants were given a diary to record awakening time, the times their samples were supposed to be collected, and the time when they actually took them. In this diary, they also reported the time they went to bed the night prior to collection. Adherence to the saliva sampling protocol seemed to be excellent. According to patients’ reports, no sampling time deviated more than 5 min from the requested saliva collection times relative to reported awakening.

Samples were never collected on the days when cognitive measures or physical performance measures were performed.

#### Physical performance

In order to measure physical performance four tests included in the Senior Fitness Test [32] were used: the 8-Meter Walking Test (8MWT) as a reflection of basic mobility and walking speed; the 6-Minute Walk Test (6MWT) to assess daily life functional exercise; the 30-Seconds Chair Stand Test (30SCST) to measure lower body strength; and the Timed Get Up & Go Test (TGUG) to measure dynamic balance and functional mobility.

#### Participant characteristics

Age, sex and education of participants were recorded. The educational score was obtained by giving participants a score of 1 to 4 as follows: 1 = no formal studies finished (reading and writing); 2 = primary education finished; 3 = secondary education finished; 4 = university education finished. Participants were also scored for depression by the Geriatric Depression Scale (GDS). These potential confounders were taken into account during statistical analysis.

### Data preparation

Based on our measures we calculated multiple indices to describe detailed aspects of cognitive function, the diurnal cycle of cortisol and physical performance. Unless otherwise indicated, original data were natural log transformed when they were characterized by a skewed nature.

#### Cognitive data

The following formula was used to transform the scores of the MMSE: √30-MMSE_score_. [33]. A lower value for the resulting index (*MMSE*) corresponds with better general cognitive function.

The time to complete TMT-A (*TMT-A*_*t*_) and TMT-B (*TMT-B*_*t*_) represented the first cognitive indices corresponding the TMT. Additionally, two indices were derived: *TMT*_*B-A*_ was calculated by subtracting TMT-A_t_ from TMT-B_t_ and *TMT*_*B/A*_ calculated by dividing *TMT-B*_*t*_ by *TMT-A. TMT-A*_*t*_ mainly reflects visuoperceptual abilities and *TMT-B*_*t*_ primarily represents working memory and secondarily mental flexibility [31, 34]. *TMT*_*B-A*_ is meant to be an indicator of executive control abilities by eliminating the speed component [31, 35]. *TMT*_*B/A*_ is thought to eliminate the speed component to a greater extent than *TMT*_*B-A*_ and was suggested to be a more reliable index of executive function [36]. Concerning these indices, a lower value reflects better aspects of cognitive function.

#### The diurnal cycle of cortisol

Raw cortisol data were winsorised to two standard deviations to reduce the impact of outliers (4%), and except for T1 and T2, we applied interpolation to treat missing data (0.6%). Subsequently, data (CORT1 to CORT5) were used to calculate multiple cortisol indices. First, the cortisol awakening response (*CAR*) was calculated by subtracting CORT1 from CORT2. Second, an aspect of diurnal decline or fall in cortisol levels after the peak *(Fall*) was calculated by subtracting CORT3 from CORT2. The mean level of cortisol (*Mean*_*All*_) was calculated based on the average of CORT1, 2, 3, 4 and 5. A second mean level of cortisol (*Mean*_*PostCAR*_) was calculated as an average of CORT3, 4 and 5. The variance in cortisol levels (*Variance*_*All*_) was calculated based on the variance of CORT1, 2, 3, 4 and 5. A second variance (*Variance*_*PostCAR*_) was determined based on the variance among CORT3, 4 and 5. Eventually, six different cortisol indices were calculated and used in the analysis. All these indices were used in former studies and are believed to be an adequate reflection of the diurnal cycle of cortisol [10, 37].

#### Physical performance

Results on the 8MWT and the TGUG are expressed as a time needed to perform the test. A higher value for these indices, *8MWT* and *TGUG*, corresponds with a lower physical performance level. Results on the 6MWT and the 30SCTS are expressed as a distance and a number of repetitions, respectively. A higher value for *6MWT* and *30SCST* corresponds with a higher physical performance level.

### Statistical analyses

#### Descriptive statistics

Categorical variables were compared using Pearson’s chi-square tests and continuous variables were compared using ANOVA and Mann–Whitney U tests for normally and non-normally distributed data, respectively. Subsequently, ANCOVA was performed to investigate if observed differences between groups held significance when controlling for the potential confounders, sex, age, education and depression.

#### Within-group analysis

Normal distribution of data was checked with the Shapiro–Wilk test. Correlations among the three outcomes were explored using Pearson and Spearman correlations coefficients. Multiple regression analysis was performed in various phases to investigate the mutual associations among the three outcomes. Subsequently, we conducted another round of multiple regression analyses using the indices which showed a significant association with both other type of indices to investigate the potential mediating role of cortisol indices in the relationship between physical performance and cognitive function. The amount of explanation was defined as the relative change in the regression coefficient of the physical performance index when including a cortisol index as an additional independent variable in the model [38]. First, data of both groups were combined and analyzed as one group. Then, the aMCI group and the healthy group were analyzed individually. All regression models were controlled for sex, age, education and depression. The assumptions of normality, linearity and homoscedasticity were assessed using residual plots and were met for all regression models.

All analyses were performed using a 2.2 Windows version of the SPSS statistical package (SPSS Inc., Chicago, IL, USA). *P*-values were based on two-sided tests and significance level was set at 0.05 for all analyses.

## Results

### Descriptive statistics

The characteristics of the entire sample showing the differences between the aMCI group and the healthy group are presented in Table 1. The aMCI group was significantly older than the healthy group and had a significantly lower level of education. The proportions of male and female participants were identical in both groups.

**Table 1:** Overview of participant characteristics and the differences between the aMCI group and the healthy group

	All participants (*n* = 60)		aMCI (*n* = 30)		Healthy (*n* = 30)			
	N or Mean	% or SD	N or Mean	% or SD	N or Mean	% or SD	P	
Sex (female)	32	53.3	16	53.3	16	53.3	1.00^a^	
Age (years)	70.6	5.5	72.3	5.4	68.7	4.1	.007^b,*^	
Education (score)	2.5	1.1	2.1	1.1	2.9	.9	.013^b,*^	
Depression (score)	4.7	3.6	4.1	2.4	5.3	4.5	.556^b^	
MMSE (score)	28.6	5.3	24.5	3.9	28.4	1.9	.000^c,**^	.000^d,*^
TMT-A_t_ (s)	79.9	53.6	111.9	59.2	49.0	19.1	.000^b,**^	.000^d,**^
TMT-B_t_ (s)	189.0	145.4	308.1	173.9	118.4	53.0	.000^c,**^	.000^d,**^
TMT_B-A_ (s)	126.8	133.5	226.7	179.1	71.2	43.0	.000^c,**^	.000^d,**^
TMT_B/A_	3.1	2.0	4.2	2.9	2.5	0.9	.044^c,*^	.101^d^
CAR	.24	.43	.33	.46	.17	.39	.216^c^	
Fall	.87	.91	.83	.51	.91	.45	.609^c^	
Mean_PostCAR_	1.42	.37	1.43	.35	1.41	.40	.884^b^	
Mean_All_	1.88	.35	1.84	.31	1.92	.38	.439^c^	
Variance_PostCAR_	.30	.20	.29	.18	.30	.22	.887^b^	
Variance_All_	.63	.30	.56	.24	.69	.34	.119^c^	
8MWT (s)	5.6	1.7	6.6	1.7	4.5	.1	.000^c,**^	.000^d,**^
6MWT (m)	504.9	87.8	442.2	54.0	569.8	66.3	.000^c,**^	.000^d,**^
30SCST (score)	14.0	4.1	11.2	2.5	17.2	3.3	.000^c,**^	.000^d,**^
TGUG (s)	6.7	2.0	8.0	1.8	5.3	.8	.000^c,**^	.000^d,**^

Categorical variables and continuous variables were tabulated as absolute numbers and percentages and as means and standard deviations (SD), respectively. *P*-values refer to the level of significance when comparing the aMCI group and the healthy group. Original scores from the cognitive measures and physical performance measures and their derivations were tabulated. Transformed indices were used to compare the aMCI group and the healthy group, without controlling (^b,c^) and with controlling for covariates, sex, age, education and depression (^d^)

^a^Chi square test

^b^Mann–Whitney U test

^c^ANOVA

^d^ANCOVA

^*^Significant at the .05 level

^**^Significant at the .01 level

Healthy participants scored significantly better for all cognitive indices. Except for TMT_B/A_, all the differences held significance after controlling for covariates, sex, age, education and depression. No significant differences were observed between groups for the different cortisol indices. Healthy participants scored significantly better on all four physical performance measures, and all the differences held their significance after controlling for covariates, sex, age, education and depression.

### The relationship between cognitive indices and physical performance indices

Significant positive correlations were observed between MMSE and 8MWT, TGUG indicating that better cognition was associated with faster walking speed and better dynamic balance and functional mobility (Table 2). MMSE showed significant negative correlations with 6MWT and 30SCST, indicating that better cognition was associated with higher daily life functional exercise capacity and lower body strength. TMT-A_t_ was found to be significant positively correlated with 8MWT and TGUG and significant negatively correlated with 6MWT and 30SCST. TMT-B_t_ showed significant positive correlations with 8MWT and TGUG and significant negative correlations with 6MWT and 30SCST. TMT_B-A_ was found to be significant positively correlated with 8MWT and TGUG and significant negatively correlated with 6MWT and 30SCST. These results indicated that better executive function was associated with higher walking speed, dynamic balance, daily life functional exercise capacity, and lower body strength.

**Table 2:** Overview of the significant correlations and associations between indices for the entire sample

Dependent variable	Independent variable	Correlation		Regression					
		r	P	Adj. R^2^	∆R^2^	F	P	β	P
MMSE	8MWT	.733	.000^b^	.528	.261	12.855	.000^b^	.621	.000^b^
	6MWT	−.743	.000^b^	.587	.314	16.040	.000^b^	−.639	.000^b^
	30SCST	−.733	.000^b^	.566	.297	14.574	.000^b^	−.615	.000^b^
	TGUG	.770	.000^b^	.590	.318	16.285	.000^b^	.691	.000^b^
	Variance_All_	−.315	.023^a^	.302	.061	5.330	.001^a^	−.254	.043^a^
TMT-A_t_	8MWT	.589	.000^b^	.350	.096	6.383	000^b^	.377	.009^b^
	6MWT	−.630	.000^b^	.378	.120	7.082	000^b^	−.398	.003^b^
	30SCST	−696	.000^b^	.499	.232	10.544	000^b^	−.544	.000^b^
	TGUG	.720	.000^b^	.429	.168	8.526	000^b^	.502	.000^b^
TMT-B_t_	8MWT	.391	.010^a^	.233	.127	3.489	.011^a^	.433	.013^a^
	6MWT	.447	.003^b^	.274	.163	4.090	.005^a^	−.460	.004^b^
	30SCST	^.^470	.002^b^	.306	.195	4.440	.003^b^	−.499	.002^b^
	TGUG	.484	.001^b^	.324	.207	4.931	.002^b^	.558	.001^b^
TMT_B-A_	8MWT	.628	.000^b^	.360	.194	5.284	.001^b^	.535	.002^b^
	6MWT	−.617	.000^b^	.356	.190	5.198	.001^b^	−.497	.002^b^
	30SCST	−.575	.000^b^	.314	.158	4.295	.004^b^	−.450	.007^b^
	TGUG	.698	.000^b^	.451	.272	7.243	.000^b^	.640	.000^b^
8MWT	Variance_All_	−.316	.024^a^						
6MWT	Variance_All_	.332	.017^a^	.046	.080	1.484	.214	.322	.047^a^

Adj. *R* ^2 =^ adjusted R^2^, ∆R^2^ = R^2^ change

^a^Significant at the .05 level

^b^Significant at the .01 level

Multiple regression analysis showed significant independent associations between MMSE and 8MWT, 6MWT, 30SCST and TGUG (Table 2). Furthermore, TMT-A_t_, TMT-B_t_ and TMT_B-A_ were found to be significantly independently associated with 8MWT, 6MWT, 30SCST and TGUG.

### The relationship between cognitive indices and cortisol indices

A significant negative correlation was observed between MMSE and Variance_All_ (Table 2), indicating that better cognitive performance was associated with greater variation in cortisol secretion across the diurnal period. In multiple regression analysis the significant association between MMSE and Variance_All_ was sustained (Table 2).

### The relationship between cortisol indices and physical performance indices

Variance_All_ showed a significant negative and a significant positive correlation with 8MWT and 6MWT, respectively (Table 2) indicating that the diurnal variation in cortisol secretion predicted walking speed and daily life functional exercise capacity. Additionally, a trend towards a significant correlation was observed between Variance_All_ and TGUG (*r* =−.264, *p* = .061), suggesting a similar association between greater diurnal variation in cortisol and dynamic balance and functional mobility. Other trends were observed for correlations between CAR and 6MWT (*r* =−293, *p* = .060), 30SCST (*r* =−.270, *P* = .092) indicating that a greater CAR was associated with weaker physical performance.

Multiple regression analysis indicated that the significant association between Variance_All_ and 6MWT was preserved in the presence of potentially confounding variables (Table 2). A trend towards significance was observed for an association between Variance_All_ and 8MWT (adjusted *R* ^2^ = .038, *R* ^2^ change = .072 F = 1.395, *P* = .244, β =−.327, *P* = .059). Furthermore, CAR showed a trend towards significant associations with 8MWT (adjusted *R* ^2^ = .226, *R* ^2^ change = .174 F = 2.312, *P* = .109, β = .452, *P* = .064) and 6MWT (adjusted *R* ^2^ = .236, *R* ^2^ change = .182 F = 2.387, *P* = .101, β =−.472, *P* = .058).

### The explaining role of cortisol indices on associations between physical performance and cognitive function

The influence of Variance_All_ on the regression coefficient in the relationship between physical performance and cognition (8MWT, 6MWT and TGUG and MMSE) for all participants was explored. Comparison of β-values with their confidence intervals (CI) did not confirm a significant mediating role of Variance_All_ in the relationship between the physical performance indices and MMSE (Table 3).

**Table 3:** Overview of the explaining role of Variance_All_ on the relationship between MMSE and physical performance indices for the entire sample

		MMSE							
	R^2^	Adjusted R^2^	F	B	P	B	P	∆β (%)	95% CI
8MWT	.584	.528	10.309	2.046	.000^**^	1.920	000^**^	6.1	−70.0;82.3
6MWT	.633	.583	12.655	−3.352	.000^**^	−3.194	.000^**^	4.7	−60.5;70.0
TGUG	.645	.597	13.341	2.322	.000^**^	2.205	.000^**^	5.0	−51.6;61.7

^**^Significant at the .01 level

## Discussion

This study is one of the first to analyze cognitive function, the diurnal cycle of cortisol secretion and fitness parameters together in a sample of older adults with and without cognitive impairment.

### Between-groups differences

The initial comparison between the characteristics of the healthy older adults group and the aMCI group showed expected lower levels of cognitive function and physical performance for the aMCI group. These results are in line with the formed observed associations between development of MCI and aspects of physical performance, including dynamic balance [39] and walking speed [40].

No differences were found in regards to the cortisol indices between the two groups. A relationship between aspects of the diurnal cycle of cortisol and incidence of MCI and AD has been suggested in several former studies [41–46] and it seems that abnormalities in cortisol concentrations might trigger development of MCI and dementia [47]. However, we were not able to confirm this hypothesis based on these results.

### Physical performance and cognitive function

Physical performance was associated with cognitive function in older adults, even after controlling for sex, age, education and depression. Similar results were observed in several earlier studies in healthy older adults [48–54] and older adults with MCI [54–57]. Nevertheless, other studies in healthy older adults observed associations between aerobic fitness and aspects of executive function [52], memory [49] and between dynamic balance and general cognitive function [48], or executive function [48, 58]. Studies in older adults with MCI indicated associations between executive function, working memory and walking speed [54, 55]. Although we are not able to provide conclusions about causalities, our results are in line with the idea that maintenance of an active lifestyle can have beneficial effects on cognitive function in both healthy older adults and older adults with MCI.

### The diurnal cycle of cortisol and cognitive function

The results indicated that a greater variance in cortisol levels across the day from morning to evening was associated with better cognition, after controlling for sex, age, education and depression. Earlier studies have found associations between a slower decline in cortisol levels during the day [8, 10], a lower magnitude of the cortisol awakening response (CAR) [10], higher daily mean levels of cortisol [37] and lower general cognitive function among healthy older adults. Healthy older adults with better executive functions showed an earlier CAR [9], a greater magnitude of the CAR [8] and lower mean levels of cortisol [37] in other studies. However, these studies did not include people with cognitive impairment. The only study including cognitive impaired older adults showed that morning cortisol levels were correlated with visual memory in healthy older adults and people with aMCI, but not in people with Alzheimer’s disease [59]. In this study, cortisol was measured only once 2 h after awakening, which can give an incomplete picture of this relationship. Thus, our study is the first cross-sectional study showing a relationship between the dynamics of cortisol secretion and cognition in a sample including people with cognitive impairment.

The present study did not find a relationship between the CAR and cognition. A recent study suggested an inverted U-shaped relationship between the CAR and spatial memory [60] which might partially explain the lack of associations observed in our study in this regard.

The exact relationship between cognitive function and the diurnal cycle of cortisol is not yet clarified but the results presented here support the hypothesis that a more dynamic cortisol secretion pattern (with more variability) is indicative of a healthier profile, allowing for a better adaption response to aging and cognitive decline. On the contrary, flatter diurnal cortisol slopes seem to be associated with more rigid profiles and lower adaptation capacity [8–10]. This is in line with the evidence showing that a more dynamic cortisol secretion pattern is associated with greater synaptic plasticity [11]. There could be a parallel with Heart Rate Variability [10], a dynamic autonomic measure also closely related to stress, in which more variability indicates a healthier cardiovascular profile [61] and better adaptive cognitive functioning [62].

### Physical performance and the diurnal cycle of cortisol

In addition, the results indicated an association between physical performance and the diurnal cycle of cortisol in older adults, even after controlling for sex, age, education and depression. The results again indicate the importance of the variance in cortisol levels during the day in this relationship. Other studies observed associations between slower walking speed and a slower decline in cortisol levels during the day [23, 24] and higher levels of cortisol [25], between greater lower body strength and a faster decline in cortisol levels [24], and between inferior lower body strength and higher levels of cortisol in healthy older adults [25]. The physical tests used in these studies were walking speed, the chair rise (5 times), standing balance, and hand grip strength. They did not consider a measure of cardiovascular and functional capacity such as the 6MWT. In our study, higher variance in cortisol diurnal levels corresponded with a better score in the 6MWT. This test is easy to administer and can be used in both healthy and cognitive impaired older adults. Thus, future studies should consider including this physical fitness test. In parallel, these studies only included healthy older adults. The results of our study possibly indicate that the observations made in healthy older adults about a relationship between better physical performance and a more dynamic cortisol secretion pattern are also sustained for people with aMCI, but further research is needed to clarify this.

### The role of the diurnal cycle of cortisol for PA-induced benefits on cognitive function

Our results showed that the same measure of variance in diurnal cortisol secretion was related with physical and cognitive performance. Similarly, two fitness tests (walking speed and the 6MWT) were correlated with both cognition and cortisol levels. However, our results could not confirm statistically such relationship.

Some limitations of the study should also be noted. The sample size and the cross-sectional nature of the study do not allow for generalizations. Second, due to the nature of our cortisol sampling protocol, the cortisol awakening response (and other aspects of the diurnal cycle of cortisol) [63] might be biased by using of a single sampling day. However, the diurnal rhythm of cortisol has also been shown to display relative intra-individual stability between days and have thus shown good reliability using a single sampling day in other studies [7].

## Conclusions

Our study on older adults is one of the first in the field to combine data for cognitive function, the diurnal cycle of cortisol and physical performance in one analysis, with a sample of older adults with and without cognitive impairment. Our results are in line with the hypothesis that physical activity has beneficial effects on cognitive function in healthy older adults and older adults with aMCI. Second, the results support the idea that a more dynamic cortisol secretion pattern is associated with better cognitive function and physical performance even in the presence of cognitive impairment. However, the main purpose of this study was to investigate if the diurnal cycle of cortisol mediates the possible beneficial effects of physical activity on cognitive function. The results of this study could not confirm statistically such a relationship in this population of healthy older adults and older adults with aMCI. Future studies should attempt to repeat our findings with larger sample sizes, and ideally collecting cortisol samples on multiple days. They should also consider selecting samples only with cognitive impairment. Furthermore, they should collect longitudinal data in order to reveal causal relationships among cognitive function, the diurnal cycle of cortisol and physical performance.

## Abbreviations

30SCST: 30-Seconds Chair Stand Test
6MWT: 6-Minute Walk Test
8MWT: 8-Meter Walking Test
AD: Alzheimer’s disease
aMCI: Amnestic Mild Cognitive Impairment
CAR: The cortisol awakening response
GDS: Geriatric Depression Scale
HPA: Hypothalamic pituitary axis
MCI: Mild Cognitive Impairment
MMSE: Mini-Mental State Examination
PA: Physical activity
TGUG: Timed get up & go test
TMT: Trail making test
